# Neurocognitive Functioning in Schizophrenia and Bipolar Disorder: Clarifying Concepts of Diagnostic Dichotomy vs. Continuum

**DOI:** 10.3389/fpsyt.2013.00162

**Published:** 2013-12-05

**Authors:** Carissa N. Kuswanto, Min Y. Sum, Kang Sim

**Affiliations:** ^1^Research Division, Institute of Mental Health/Woodbridge Hospital, Singapore; ^2^Department of General Psychiatry, Institute of Mental Health/Woodbridge Hospital, Singapore

**Keywords:** schizophrenia, bipolar disorder, Kraepelinian dichotomy, neurocognitive functions, psychosocial functioning, psychotic spectrum

## Abstract

The Kraepelinian dichotomy posits that patients with schizophrenia (SCZ) and bipolar disorder (BD) present as two separate psychotic entities such that they differ in terms of clinical severity including neurocognitive functioning. Our study aimed to specifically compare and contrast the level of neurocognitive functioning between SCZ and BD patients and identify predictors of their poor neurocognitive functioning. We hypothesized that patients with SCZ had a similar level of neurocognitive impairment compared with BD. About 49 healthy controls (HC), 72 SCZ, and 42 BD patients who were matched for age, gender, and premorbid IQ were administered the Brief Assessment of Cognition battery (BAC). Severity of psychopathology and socio-occupational functioning were assessed for both patients groups. Both BD and SCZ groups demonstrated similar patterns of neurocognitive deficits across several domains (verbal memory, working memory, semantic fluency, processing speed) compared with HC subjects. However, no significant difference was found in neurocognitive functioning between BD and SCZ patients, suggesting that both patient groups suffer the same degree of neurocognitive impairment. Patients with lower level of psychosocial functioning [*F*_(1,112)_ = 2.661, *p* = 0.009] and older age [*F*_(1,112)_ = −2.625, *p* = 0.010], not diagnosis or doses of psychotropic medications, predicted poorer overall neurocognitive functioning as measured by the lower BAC composite score. Our findings of comparable neurocognitive impairments between SCZ and BD affirm our hypothesis and support less the Kraepelinian concept of dichotomy but more of a continuum of psychotic spectrum conditions. This should urge clinicians to investigate further the underlying neural basis of these neurocognitive deficits, and be attentive to the associated socio-demographic and clinical profile in order to recognize and optimize early the management of the widespread neurocognitive deficits in patients with SCZ and BD.

## Introduction

The Kraepelinian dichotomy, a prominent paradigm in psychiatry, has influenced nosology for severe psychiatric disorders such as schizophrenia (SCZ) and bipolar disorder (BD) within diagnostic classification systems including the Diagnostic Statistical Manual of Mental Disorders and the International Classification of Diseases for many decades (1, 2). The paradigm posits that patients with dementia praecox (SCZ) and manic depression (BD) present as two separate psychotic disorders (3, 4). Evidence in support of the Kraepelinian notion includes volumetric neuroimaging differences in brain regions such as the amygdala, hippocampus, and lateral ventricles that appear to be disorder-specific (5–7). In addition, unlike BD, reduced gray matter volumes and neurocognitive neuromotor impairments occur in the prodromal stage of SCZ despite both having early onset of illness during adolescence and recent findings of common genetic predisposition in BD and SCZ (6, 8–13). Furthermore, patients with SCZ are thought to suffer more extensive brain morphological abnormalities and more severe neurocognitive deficits in comparison with patients with BD (14–18). Other than the apparent empirical evidence, the Kraepelinian dichotomy is appealing clinically due to its conceptual and diagnostic simplicity (19, 20).

However, several issues challenge the dichotomous paradigm of psychotic disorders with respect to SCZ and BD. These issues include the overlapping organic basis and blurred boundaries between psychoses from emerging biological data (21, 22), the non-specificity of psychopathological features such as perceptual disturbances and affective symptoms between SCZ and BD, and the shared genetic susceptibility loci uncovered within recent genome-wide association studies (GWAS) (23, 24). Accumulating evidence from clinical observations, antipsychotic treatment, and findings of neurocognitive functioning in SCZ and BD further argued against the Kraepelinian dichotomy. First, clinicians frequently encounter patients who do not fall neatly into either category, such as individuals with schizoaffective disorders and BD patients with prominent psychotic symptoms. Second, dopamine dysregulation has been implicated in both disorders, making antipsychotic drugs useful in the management of SCZ as well as BD (6, 8). Third, with regards to neurocognitive functioning, extant studies have reported poorer neurocognitive functioning in patients with SCZ compared to those with BD, but there are also data suggesting that patients with SCZ are comparable with BD patients in terms of neurocognitive impairments (14, 25–31). These inconsistent findings on neurocognitive functioning may be due to differences in sample size, specific neurocognitive deficits measured, type of scales used, and presence of other confounders including age and premorbid intelligence (26, 27, 30, 32).

Thus, in this study, we aimed to specifically compare and contrast the level of neurocognitive functioning between patients with SCZ and BD as well as identify predictors of poor neurocognitive functioning in these patients. Based on clinical impression within a tertiary psychiatric hospital context and extant data, we hypothesized that patients with SCZ had a similar level of neurocognitive impairment compared with BD and the neurocognitive impairment is associated with particular socio-demographic and clinical factors after taking into account premorbid intelligence, level of education, and psychotropic medications prescribed.

## Materials and Methods

### Participants

The cross-sectional study sample comprised of a total of 163 participants [72 patients diagnosed with SCZ, 42 patients with BD, and 49 healthy controls (HC)] who gave their written informed consent to participate in the study after a detailed explanation of the study procedures. Patients with SCZ and BD were recruited from the outpatient settings of the Institute of Mental Health, Singapore. All diagnoses were confirmed by a psychiatrist (Kang Sim) using information obtained from the existing medical record, clinical history, mental status examination, interviews with the patients and their significant others as well as the administration of the Structured Clinical Interview for DSM-IV disorders – Patient Version (SCID-I/P) (33). All patients were maintained on a stable dose of psychotropic medication for at least 2 weeks prior to the recruitment and did not have their medication withdrawn for the purpose of the study. HC were administered the Structured Clinical Interview for DSM-IV disorders – Non-Patient Version (SCID-I/NP) (34). None of the participants had a history of significant and/or unstable/untreated medical illnesses such as seizure disorder, head trauma, or cerebrovascular accidents. Moreover, no subjects had a current or past history of substance use or alcohol use disorder. This study was approved by the Institutional Review Boards of the Institute of Mental Health, Singapore.

### Neurocognitive assessment

#### The brief assessment of cognition battery

The Brief Assessment of Cognition (BAC) battery (35) was administered to all subjects. The subjects were randomly assigned to a sequence of BAC version A and B. The BAC scales include:

1. Verbal memory task – a subject was given 5 attempts to remember 15 words and recall as many words as possible;
2. Digit sequencing task – a subject was presented with clusters of numbers of increasing length and then asked to repeat in order from the lowest to highest length;
3. Token motor task – a subject was given 100 plastic tokens and asked to place 2 tokens at a time within a container as quickly as possible within 60 s;
4. Verbal fluency – divided into semantic and letter fluency, whereby a subject was asked to name as many words as possible within a specific category (e.g., supermarket items), and to name words that begin with a specific letter (e.g., F and S) within 60 s, respectively;
5. Symbol coding task – a subject was asked to write matching numbers from 1 to 9 to symbols within 90 s;
6. Tower of London – as an example, a subject was shown two pictures of three balls of different colors arranged on three different pegs, whereby the balls were arranged differently on each picture and the subjects were asked to give the total number of times the balls in one picture needed to be moved in order to end with the arrangement in the other picture.

We divided the token motor task into two measures, namely the number of tokens correctly placed into a container (tokens correct) and the number of tokens left after 60 s (tokens left). Although both measures tap motor speed function, tokens correct measure did not credit the subjects for tokens put into container incorrectly (i.e., not picked up and placed at the same time) which required eye-hand coordination and precision in order to perform the task correctly. Digit sequencing task, token motor task, symbol coding, and Tower of London measured working memory, motor speed, attention, and speed of information processing and executive functioning respectively (35, 36).

#### Wide range achievement test (reading scale)

The wide range achievement test (WRAT) (37) reading subscale was used to measure premorbid intelligence in all the subjects.

### Clinical assessments

#### Global assessment of functioning

The global assessment of functioning (GAF) (38) rated (from 0 to 90) the severity of symptoms, disability, and the total level of social and occupational functioning. The GAF was administered to both SCZ and BD groups.

#### Positive and negative syndrome scale

The positive and negative syndrome scale (PANSS) (39) allowed characterization and quantification of psychotic psychopathology. The scale has 7 items for positive symptoms, 7 items for negative symptoms, and 16 items for general psychopathology. The PANSS was administered to SCZ and BD patients.

#### Young mania rating scales

The Young mania rating scales (YMRS) (40) contains 11 items which were used to measure the severity of manic symptoms in BD patients. There are four items that are graded on a 0–8 scale (irritability, speech, thought content, and disruptive/aggressive behavior). The remaining seven items are graded on a 0–4 scale. The YMRS scale was administered to SCZ and BD patients.

### Statistical analyses

All statistical tests were performed using PASW for Windows, version 18.0 (SPSS, Inc., Chicago, IL, USA). The normality of distributions of continuous measures was checked with the Kolmogorov–Smirnov one-sample test. Socio-demographic variables were compared using two sample student *t*-tests and *F*-test for continuous variables, and chi-square test for categorical variables. Two-way analysis of covariance (ANCOVA) was used to examine for any difference in neurocognitive functioning (BAC task performance) between the groups after adjusting for the covariates described below. We first compared the neurocognitive measures between all three groups, HC vs. SCZ and HC vs. BD, and adjusting for covariates of age, gender, years of education, and premorbid IQ. We then specifically compared the BAC item performance between SCZ and BD patients, by adjusting for premorbid IQ, age, gender, years of education, and clinical characteristics such as the duration of illness, duration of untreated psychosis, GAF scores, PANSS composite scores, YMRS scores, and antipsychotic dose in terms of mean daily chlorpromazine (CPZ) mg equivalents. Stepwise linear regression analyses were performed to determine predictors of neurocognitive functioning in patients including socio-demographic (such as age, gender) and clinical characteristics (such as diagnosis, duration of illness, GAF scores, PANSS composite scores, YMRS scores, and antipsychotic dose). The significance level for statistical tests was set at two tailed *p* < 0.05.

## Results

### Socio-demographic characteristics

Across the three subject groups (HC, SCZ, BD), there were significant differences in subject’s and mother’s years of education but there was no significant difference in age, sex, handedness, and WRAT scores. Compared to patients with BD, patients with SCZ had longer duration of untreated psychosis and lower level of psychosocial functioning as reflected by the lower GAF scores. There was no significant difference in age, gender, handedness, years of education, WRAT scores, age of onset, and duration of illness between SCZ and BD. The socio-demographic and clinical features of the subjects are shown in Table 1.

**Table 1:** **Demographic and clinical characteristics of the participants**.

Characteristic	HC (*n* = 49)		SCZ (*n* = 72)		BD (*n* = 42)		Statistical analysis			
							SCZ vs. BD		All three groups	
	Mean	SD	Mean	SD	Mean	SD	*t*-test	*p*	*F*-test	*p*
Age	31.9	10.4	31.47	8.29	32.57	9.673	−0.642	0.522	0.191	0.827
Subject’s years of education	13.59	1.62	12.31	2.039	12.15	2.291	0.364	0.717	7.849	0.001*
Father’s years of education	8.53	4.22	7.62	3.697	7.31	3.317	0.447	0.655	1.454	0.237
Mother’s years of education	7.88	4.29	6.93	3.815	4.98	4.129	2.551	0.012*	6.228	0.002*
WRAT scores	49.78	4.25	49.75	5.301	49.88	4.994	−0.13	0.897	0.75	0.474
Duration of illness	–	–	4.77	5.317	3.32	56.39	1.394	0.166	–	–
Age of onset	–	–	26.69	8.073	28.24	9.294	−0.931	0.354	–	–
Duration of untreated psychosis	–	–	1.715	2.206	0.296	0.528	4.094	<0.001*	–	–
Mean daily CPZ mg equivalents	–	–	185.76	197.47	157.38	174.89	0.771	0.442	–	–
GAF scores
Total	–	–	50.18	18.821	64.9	21.101	−3.852	<0.001*	–	–
Symptoms	–	–	54.36	20.292	64.98	21.39	−2.641	0.009*	–	–
Disability	–	–	51.46	19.092	65.69	19.749	−3.791	<0.001*	–	–
PANSS
Positive symptoms	–	–	10.79	3.715	8.55	2.839	3.379	0.001*	–	–
Negative symptoms	–	–	8.33	2.116	7.05	0.309	3.906	<0.001*	–	–
General psychopathology	–	–	20.89	3.71	17.79	1.828	5.067	<0.001*	–	–
Total score	–	–	40.01	7.135	33.38	4.120	5.506	<0.001*	–	–
YMRS	–	–	1.56	2.469	3.4	4.607	2.485	0.015*	–	–

	***N***	**%**	***N***	**%**	***N***	**%**	**χ^2^**	***p***	**χ^2^**	***p***

Male	28	57.1	38	52.8	17	40.5	1.608	0.205	2.442	0.295
Right-handed	46	93.9	70	97.2	40	97.6	1.148	0.284	1.102	0.576

BD, bipolar disorder; CPZ, chlorpromazine; GAF, global assessment of functioning; HC, healthy controls; PANSS, positive and negative syndrome scale; SCZ, schizophrenia; WRAT, wide range achievement test; YMRS, young mania rating scale. *p < 0.05.

### Neurocognitive functioning

Table 2 showed the neurocognitive profile across the entire subject sample based on the BAC battery administration. HC subjects scored the highest in all BAC items compared to SCZ and BD patients. Compared to HC, patients with SCZ scored lower in BAC tasks of verbal memory, semantic and letter fluency, digit sequencing, tokens left, symbol coding, total BAC score indicating neurocognitive deficits in verbal memory and fluency, working memory, motor speed, and attention. Compared to HC, patients with BD scored lower in BAC tasks of verbal memory, semantic and letter fluency, digit sequencing, tokens correct, symbol coding, total BAC score indicating almost similar neurocognitive deficits as SCZ in verbal memory and fluency, working memory, motor speed, and attention. After taking into account multiple comparisons (at conservative threshold of *p* < 0.005), patients with SCZ scored lower on tasks of verbal memory, semantic fluency, symbol coding, and patients with BD scored lower on verbal memory, digit sequencing, and symbol coding when compared with HC.

**Table 2:** **Neurocognitive functioning across subjects based on brief assessment of cognition battery (BAC)**.

BAC domains	HC (*n* = 49)		SCZ (*n* = 72)		BD (*n* = 42)		ANCOVA^a^						ANCOVA^b^	
							All three groups		HC vs. SCZ		HC vs. BD		SCZ vs. BD	
	Mean	SD	Mean	SD	Mean	SD	*F*	*p*	*F*	*p*	*F*	*p*	*F*	*p*
Verbal memory	9.07	1.92	7.60	2.11	7.85	1.91	6.45	0.002**	9.84	0.002**	11.99	0.001**	0.92	0.340
Digit sequencing	21.62	3.01	19.04	4.54	18.68	4.07	4.64	0.011*	7.13	0.009*	9.74	0.002**	0.05	0.942
Tokens correct	76.12	12.90	73.46	69.26	64.55	15.97	0.54	0.587	0.12	0.729	10.44	0.002**	0.63	0.430
Tokens left	19.56	12.96	25.57	12.65	26.00	13.60	3.50	0.033*	6.55	0.012*	3.90	0.051	0.32	0.573
Semantic fluency	24.64	5.82	20.10	5.77	21.17	5.86	6.13	0.003**	12.05	0.001**	5.58	0.020*	0.18	0.674
Letter fluency	13.91	3.71	12.51	4.51	12.21	4.82	4.27	0.016*	6.83	0.010*	7.35	0.008*	0.02	0.895
Symbol coding	64.68	10.43	49.21	11.42	50.55	10.45	27.47	<0.001**	48.31	<0.001**	32.85	<0.001**	0.08	0.773
Tower of London	17.04	2.12	16.17	3.79	16.27	3.49	0.24	0.785	0.36	0.548	0.17	0.683	0.08	0.783
BAC total	277.66	28.25	239.36	77.12	232.60	43.69	6.27	0.002**	8.00	0.006*	28.55	<0.001**	0.36	0.552

BAC, brief assessment of cognition battery; BD, bipolar disorder; CPZ, chlorpromazine; GAF, global assessment of functioning; HC, healthy controls; SCZ, schizophrenia; WRAT, wide range achievement test. *p < 0.05 **p < 0.01.

*^a^ Adjusted for WRAT, age, gender, and years of education*.

*^b^ Adjusted for WRAT, age, gender, years of education, duration of untreated psychosis, duration of illness, GAF (total, symptoms, and disability), antipsychotic dose, and PANSS and YMRS composite scores*.

However, there was no significant difference in all neurocognitive domains of BAC when SCZ and BD groups were compared and after controlling for covariates such as demographic characteristics (age, gender, years of education, WRAT scores) and clinical features (duration of untreated psychosis, antipsychotic dose, as well as GAF, PANSS, and YMRS composite scores). We also noted the possibility that a history of psychosis in BD patients may be associated with poorer performance in neurocognitive tasks compared to those BD subjects without history of psychosis (30). Thus, we performed a *post hoc* analysis whereby we compared BD patients with history of psychosis and those without and we found no significant difference in all neurocognitive performance in all BAC domains.

Multivariate linear regression analyses with the overall BAC score as the dependent variable and demographic (age, gender, education) and clinical factors (diagnosis, WRAT scores, GAF scores, PANSS composite scores, YMRS scores, duration of illness, duration of untreated psychosis, antipsychotic dose) as predictor variables revealed that patients with lower level of psychosocial functioning as indicated by lower GAF score [*F*_(1,112)_ = 2.023, *p* = 0.046] and older age [*F*_(1, 112)_ = −2.190, *p* = 0.031], but not diagnosis or doses of psychotropic medications, were associated with poorer overall neurocognitive functioning as measured by the lower BAC composite score.

## Discussion

There were several main findings in this study. First, patients with BD and SCZ were found to have greater neurocognitive impairments in most of the BAC domains compared to the HC who were matched for age, gender and premorbid intelligence. Second, the extensive neurocognitive impairments found in SCZ were comparable with those observed in patients with BD. Third, the level of psychosocial functioning and age of patients with SCZ and BD were associated with neurocognitive impairment and not diagnosis or antipsychotic dose. Our findings highlighted that both SCZ and BD patients in our study sample suffered largely similar neurocognitive impairments, which support a continuum concept of psychosis rather than the Kraepelinean concept of dichotomous psychotic conditions.

Our findings of comparable and extensive neurocognitive impairments in both SCZ and BD are consistent with some earlier studies reporting findings of specific neurocognitive impairments across the patient groups. For example, BD and SCZ patients scored significantly worse than control subjects in verbal memory task (assessed by the California Verbal Learning Test), which did not differ when both patient groups were compared (25, 29). Likewise, motor speed was also impaired in patients with SCZ/BD during the Grooved Pegboard test when compared with HC (14, 31). Furthermore, other studies have shown comparable impairments in domains of verbal fluency and working memory within both SCZ and BD patients (14, 25, 28). In terms of executive functioning, a number of studies found comparable neurocognitive deficits in both patient groups irrespective of the tests used such as Wisconsin Card Sorting Test (WCST) (14, 29, 41), Trail Making Test B (TMT B) (28, 31), and Tower of Hanoi (29). Overall, our findings add to the literature to support the notion of comparable and pervasive neurocognitive impairments in patients with BD and SCZ (17, 29).

What may be the biological factors that underlie SCZ and BD which may be relevant to the observed neurocognitive impairments? First, recent evidence from large-scale GWAS has pointed to possible common susceptibility genes underlying SCZ and BD which may affect neurocognitive functioning. These common vulnerability cross disorder genes include ZNF804A, CACNA1C, NRGN, and PBRM1 (5, 19, 23, 24, 42–45). A recent study from the Psychiatric Genome Consortium reported that there are 14 genetic loci associated with both SCZ and BD (46), and shared genetic effects between SCZ and BD accounted for 52% of the genetic variance in SCZ and 69% for BD in the Swedish population (44). Second, neuroimaging aberrations of brain morphology and function have been noted. For example, voxel-based morphometry studies found that there was a substantial overlap in gray matter volumetric reductions such as the prefrontal cortex, thalamus, caudate, medial temporal lobe, insula, and the anterior cingulate regions in SCZ and BD (7, 15, 47). Third, while genetic risks for SCZ and BD are associated with different gray matter changes, McDonald et al. (48) suggested that they share white matter endophenotypes with the reduction of white matter volume in the left frontal and temporoparietal regions in both SCZ and BD.

Of note, older age and poorer psychosocial functioning were found to be associated with neurocognitive impairments in our patients. These findings are in line with that of other studies whereby older age was associated with decreased gray matter volume (49) and poorer neurocognitive performance in SCZ, and which can affect psychosocial functioning (50). The lifetime history of psychotic features in BD patients has also been found to worsen subsequent neurocognitive performance such as cognitive flexibility and working memory indicating age related effects on neurocognitive functioning in BD (10, 26, 27, 30, 32, 51). However, in contrast, Gildengers et al. (52) found no difference in the rate of neurocognitive decline between BD and healthy subjects with age.

We found that antipsychotic medication dose was not associated with neurocognitive impairment in SCZ and BD in this study although there is evidence of antipsychotic medications affecting changes in brain structures and neurocognitive performance. Previous studies have reported both improvements in neurocognitive impairments with antipsychotic treatment in SCZ and BD (26) but negative impact on cognition have also been reported in BD (53, 54). In SCZ patients, some studies reported that the administration of typical antipsychotic medication provides only modest-to-moderate gains in multiple cognitive domains (55, 56), while other studies found no correlation between antipsychotic medication dose and neurocognitive functioning in BD and SCZ (9, 18, 57).

Recent data also suggest that neurocognitive deficits may potentially serve as endophenotypical trait markers for BD and SCZ (58–60). Unlike a state marker whereby neurocognitive performance may vary with and be influenced by current clinical states such as affective change and psychotic phenomenology, a trait marker remains stable throughout the illness course and may be related to genetic factors (60, 61). For SCZ, a twin study by Pardo et al. (58) noted preservative errors on the WCST not only in the SCZ twin but also within non-SCZ monozygotic co-twin suggesting a spectrum of genetic liability. Furthermore, clinical symptoms do not seem to play a role in worsening cognitive performance in ultra-high risk of psychosis subjects (62). In BD, Sobzcak et al. (59) found deficits in memory function, psychomotor performance and attention in high risk relatives suggesting potential neurocognitive trait markers for BD. In a recent longitudinal study involving BD patients, the severity of depressive symptoms did not predict change of performance in any cognitive domains and all domains remained stable over the course of 6 years (60).

There are several limitations to the study. First, our sample size was rather small and the study solely recruited patients with remitted SCZ and BD seen at the outpatient setting of a tertiary psychiatric hospital and thus the results may not be generalizable to patients within the inpatient and in non-tertiary psychiatric settings. Second, this was a cross-sectional study and did not allow observations of changes of neurocognitive functioning over the course of time. Third, the administrators of the neurocognitive tests were not blinded to the diagnoses of the subjects. Lastly, we did not examine other biological correlates such as genetic factors, neurophysiological measures but we intend to do so as a follow up study of these findings. These multimodality studies can potentially elucidate biological underpinnings or correlates of these neurocognitive impairments in SCZ and BD.

In conclusion, we found that patients with BD and SCZ suffer widespread neurocognitive deficits involving almost all the examined cognitive domains. The severity of neurocognitive deficits has been found to be significantly associated with various factors such as the level of psychosocial functioning and age but not specific diagnoses or antipsychotic medications. The underlying biological basis of these neurocognitive deficits may be related to factors common to both conditions and awaits better definition. Our findings support the dimensional concept of psychotic spectrum conditions along a continuum rather than the dichotomous concept as proposed by Kraepelin. Although DSM-V (63) still retains the existing nosological boundaries between BD and SCZ, clinicians would have to remain vigilant in managing each case of SCZ or BD by taking into account their clinical, socio-demographic factors, and assessing for the presence of specific neurocognitive impairment so as to better optimize the care and cater for more individually tailored treatment plans for these patients with potentially crippling conditions.

## Conflict of Interest Statement

The authors declare that the research was conducted in the absence of any commercial or financial relationships that could be construed as a potential conflict of interest.
